# Supplementary material for: Binding of the RNA Chaperone Hfq on Target mRNAs Promotes the Small RNA RyhB-Induced Degradation in Escherichia coli
Source: Noncoding RNA. 2021 Sep 28;7(4):64. doi: 10.3390/ncrna7040064 (PMC8544716; doi:10.3390/ncrna7040064)
Supplement: Supplementary file 1 [file ncrna-07-00064-s001.zip › ncrna-1297342-supplementary.pdf]

Binding of the RNA chaperone Hfq on target mRNAs is essential for small RNA-induced degradation, not for sRNA pairing

This PDF file includes Supplementary data:

Figures S1 to S5

Tables S1 and S2

References for SI reference citations

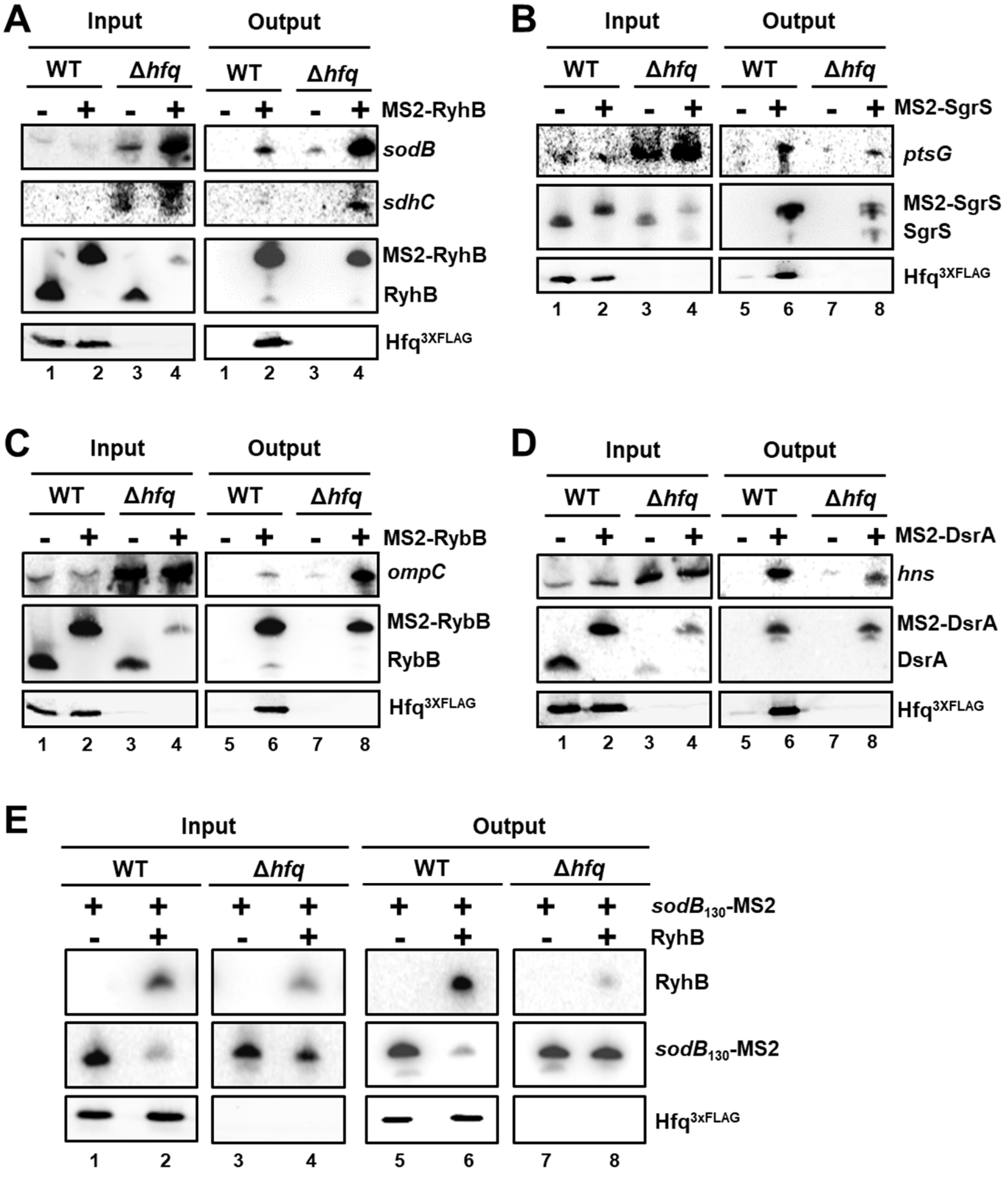

Figure S1. Hfq is not essential for the pairing between several sRNAs and their target mRNAs in vivo. (A) Visualisation of previously known target mRNAs (*sodB* and *sdhC*) after co-purification with MS2-RyhB construct in WT and Δ*hfq* strains. Untagged sRNA RyhB was used as control. The expression of both MS2-sRNA (+) and RyhB (Control; -) transcripts was induced with 0.1% arabinose for 10 min at OD_{600nm} of 0.5 (exponential phase). (B) Same as in (A) but performed with MS2-SgrS construct as bait. Visualisation of a previously known target mRNA (*ptsG*) after co-purification with MS2-SgrS

construct in WT and Δ*hfq* strains. (C) Same as in (A) but performed with MS2-RybB construct as bait. Visualisation of previously known target mRNAs (*ompC*) after co-purification with MS2-RybB construct in WT and Δ*hfq* strains. (D) Same as in (A) but performed with MS2-DsrA construct as bait. Visualisation of a previously known target mRNA (*hns*) after co-purification with MS2-DsrA construct in WT and Δ*hfq* strains. (E) Co-purification of *sodB*$_{130}$-MS2 construct with RyhB sRNA in WT and Δ*hfq* strains. The *sodB*$_{130}$-MS2 construct was cloned into a pFRΔ plasmid, under the control of *sodB* endogenous promoter. Expression of RyhB was induced at an OD_{600nm} of 0.5 by addition of 0.1% arabinose (pGD3-*ryhB* (RyhB; +)). The empty vector pGD3 was used as control (RyhB; -). Northern blots were performed with DNA probes specific for respective RNAs and anti-FLAG antibodies were used for Hfq^{3xFLAG} Western blot analysis. Results are representative of at least two independent experiments. Related to Figures 1 and 6.

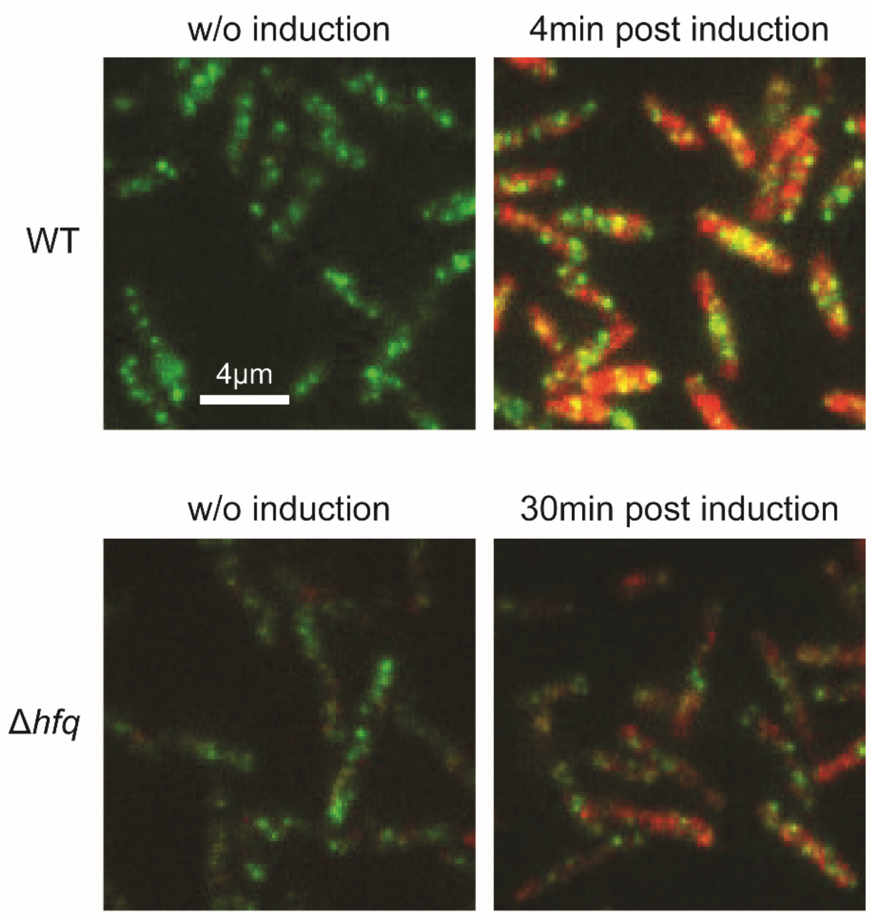

Figure S2. The level of *sodB*$_{130}$-*lacZ* construct does not change upon RyhB induction in vivo. Four representative super-resolution images are shown here. WT and Δ*hfq* cells were stained for RyhB (red, Alexa 647 dye) and *sodB*$_{130}$-*lacZ* (green, Alexa 568 dye). (Upper panels) In wild-type cells, RyhB level jumps up after 4 minutes of induction (with 250μM DIP), but *sodB*$_{130}$ level is not affected. (Lower panels) The same trend was observed in Δ*hfq* cells. RyhB was induced for 30 minutes (with 250μM DIP) since RyhB induction was slower for these cells. Related to Figure 2.

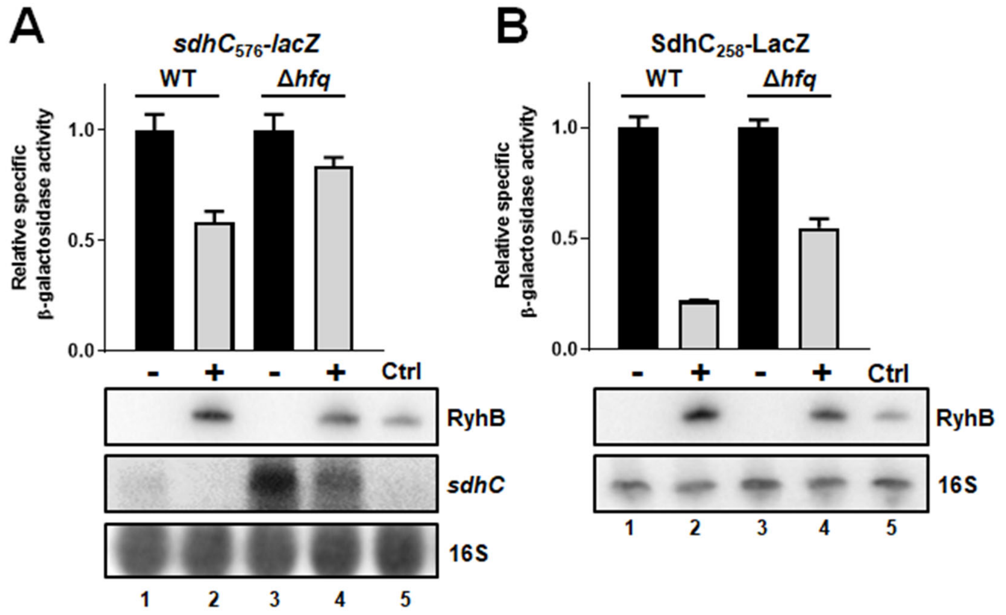

Figure S3. The absence of Hfq affects RyhB-mediated regulation of *sdhC* mRNA. β-galactosidase activity of (A) *sdhC*$_{578}$-*lacZ* transcriptional and (B) SdhC$_{258}$-LacZ translational fusions in WT and Δ*hfq* backgrounds in presence or absence of RyhB. Strains carry either an empty vector (pNM12; black) or a pBAD-*ryhB* (grey). The expression of *ryhB* was induced by addition of 0.1% arabinose when cells reached an OD$_{600nm}$ of 0.1. Samples were taken at an OD$_{600nm}$ of 0.5. Northern blot assays were performed at the same time to monitor the level of RyhB sRNA and *sdhC* mRNA. 16S rRNA was used as a loading control. As control (Ctrl, lane 5), we monitored the endogenous expression of RyhB in WT background, which was induced by addition of 250 μM DIP when cells reached an OD600nm of 0.1. Samples were taken at an OD$_{600nm}$ of 0.5. Data are representative of three independent experiments ± SD. Related to Figure 3.

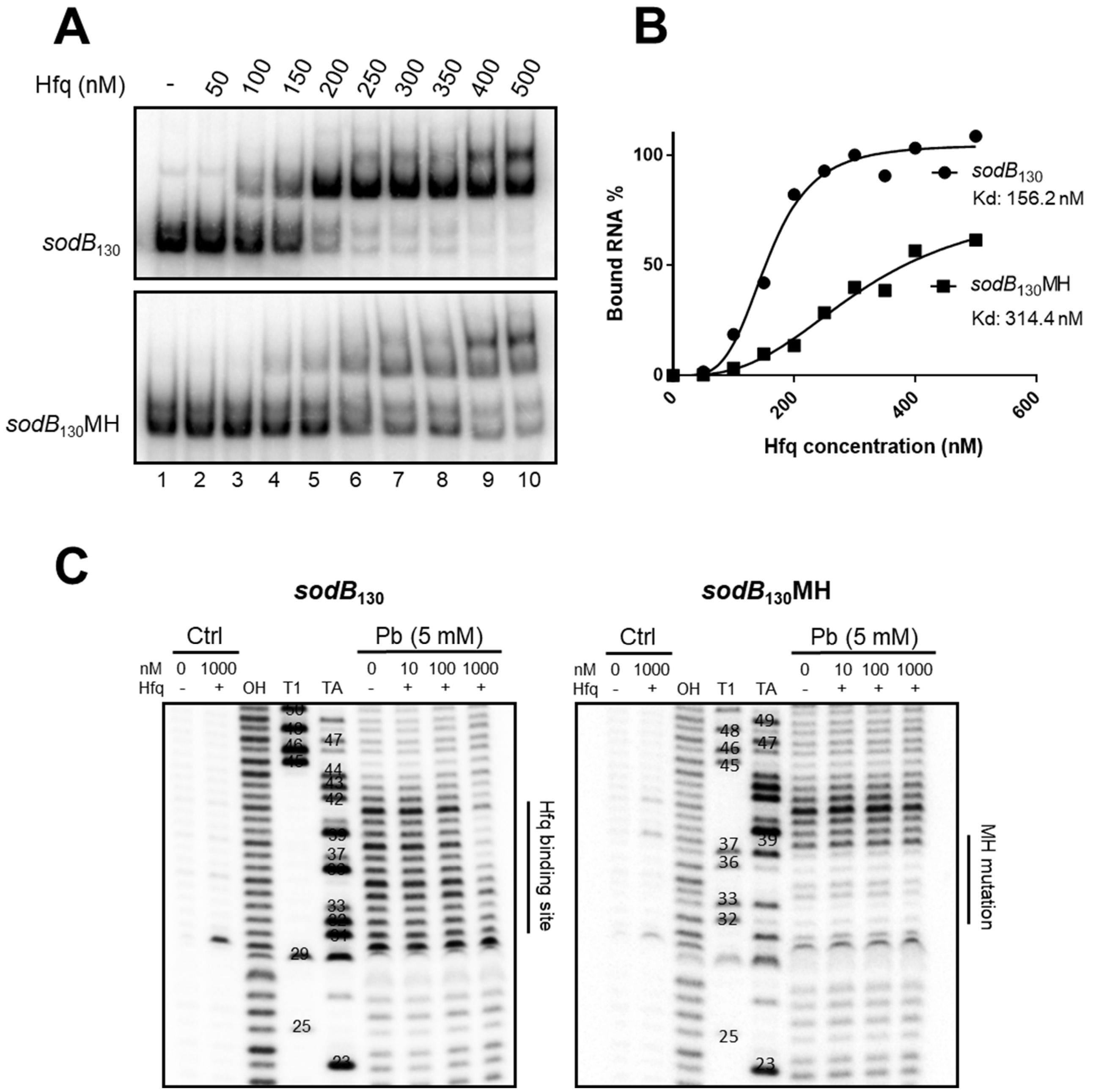

Figure S4. The mutation of Hfq binding site on *sodB* mRNA. (A) Determination of Hfq affinity by electrophoretic mobility shift assay (EMSA). Radiolabelled *sodB*130 or *sodB*130MH are incubated with increasing concentrations of Hfq hexamer (0 to 500 nM). (B) Determination of the dissociation constant (Kd) from non-linear regression of EMSA presented in Figure S4A indicating one site specific binding with Hill curve. (C) Lead acetate probing of the 5′-end-labeled *sodB*130 or *sodB*130MH mRNA in the presence of increasing amounts of Hfq (0, 10, 100 and 1000 nM). The *sodB*130MH mutation is pictured in Figure 4A. Non-reacted controls were performed in the absence (-) or presence (+) of 1000 nM Hfq. The position of several G and A residues, relative to the transcriptional start, are given. (NR) Non-reacted control; (OH) alkaline ladder; (T1) RNase T1 ladder; (TA) RNase TA ladder; (PbAc) lead acetate. Except for the ladders, all experiments were carried out under native conditions. Related to Figure 4.

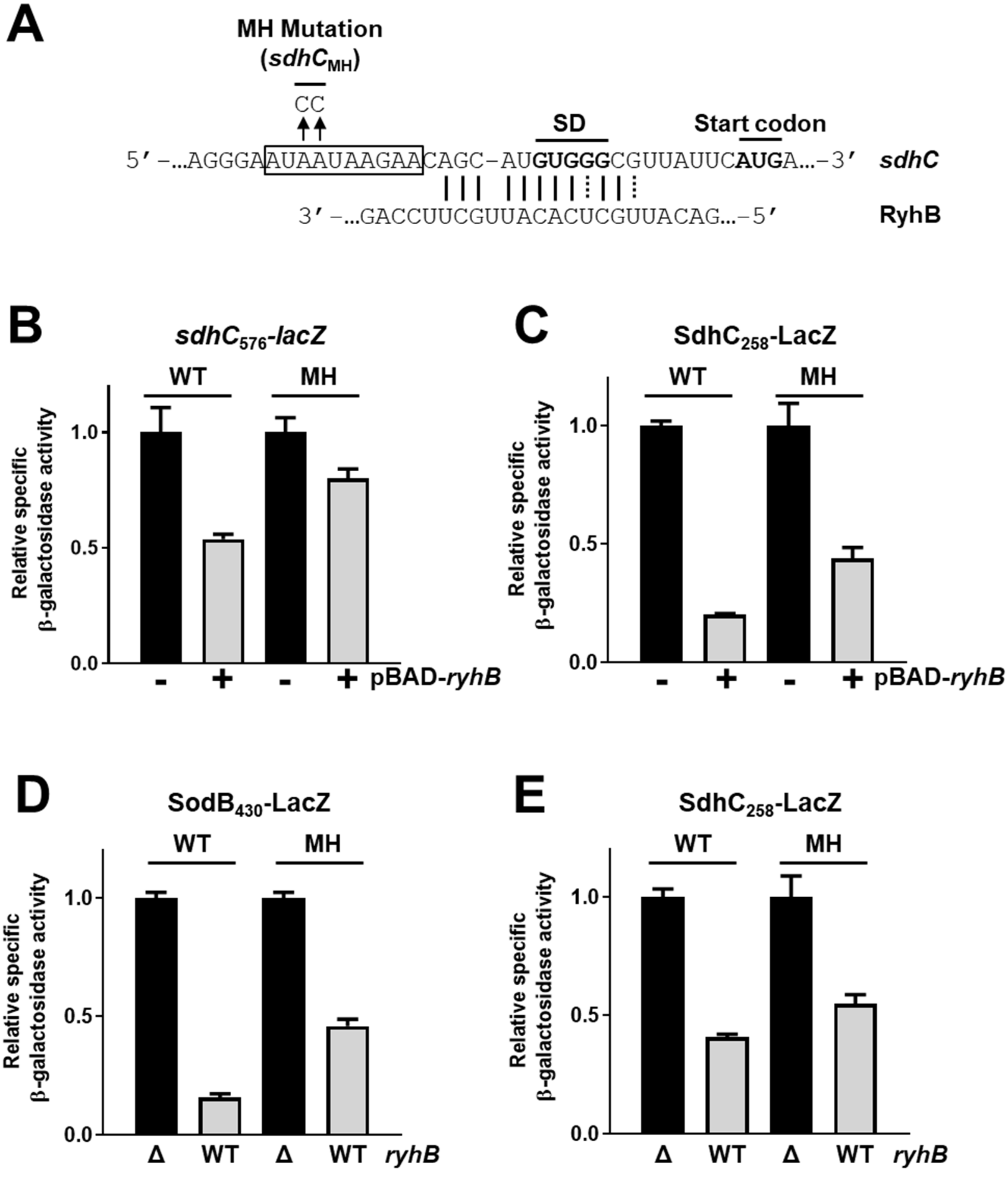

Figure S5. The Hfq binding site on *sdhC* mRNA is required for rapid RyhB-induced mRNA decay. (A) Description of RyhB sRNA binding to *sdhC* mRNA. The mutation MH located in the Hfq binding site is shown with arrows. (B) β-galactosidase activity of *sdhC*$_{576}$-*lacZ* and *sdhC*$_{576}$MH-*lacZ* transcriptional fusions in presence or absence of RyhB (pBAD-*ryhB* or pNM12). Cells were grown until an OD_{600nm} of 0.1. Then, expression of *ryhB* was induced by addition of 0.1% arabinose. Samples were taken at an OD_{600nm} of 0.5. (C) β-galactosidase activity of SdhC$_{258}$-LacZ and SdhC$_{258}$MH-LacZ translational fusions in presence or absence of RyhB (pBAD-*ryhB* or pNM12). Same procedure as in (B). Effect of RyhB sRNA on the translational SodB$_{430}$-LacZ and SodB$_{430}$MH-LacZ reporter constructs. Cells were grown until an OD_{600nm} of 0.1. Then, endogenous expression of RyhB was induced by 250μM DIP. Samples were taken at an OD_{600nm} of 0.5. (E) Effect of RyhB sRNA on the translational SdhC$_{258}$-LacZ and SdhC$_{258}$MH-LacZ fusions. Same procedure as in (D). Data are represented as mean ± SD (n=3). Related to Figure 4.

1. Supplementary Materials and Methods

Table S1. List of all strains and plasmids used in this study.

Strains	Description	Reference
EM1055	MG1655 *lacX74*	Masse and Gottesman 2002
EM1451	EM1055 Δ*ara714 leu*[+]	Desnoyers et al. 2009
EM1455	EM1055 Δ*ara714 leu*[+] Δ*ryhB*::cat	Desnoyers et al. 2009
JW4130-1	rrnB3 ΔlacZ4787 hsdR514 Δ(araBAD)567 Δ(rhaBAD)568 rph-1 Δ*hfq*-722::kan	Baba et al. 2006
KP1224	EM1055 Δ*hfq*-722::kan	EM1055 + P1(JW4130-1)
GD520	EM1055 Δ*araB*::kan	Salvail et al. 2013
GD363	EM1055 Δ*ryhB*::tet	Desnoyers et Masse 2012
GD549	EM1055 Δ*rybB*::kan	Desnoyers et Masse 2012
EM1238	EM1055 Δ*ryhB*::cat	Masse and Gottesman 2002
CS211	Δ*sgrS*::tet	C. Vanderpool Lab
DL1028	EM1055 Δ*sgrS*::tet	EM1055 + P1(CS211)
JF138	EM1055 Δ*dsrA* ::cat	Lalaouna et al. 2015a
EM1237	DY330 [W3110 ΔlacU169 gal490 λcI857 Δ(crobioA)]	Yu et al. 2000
KP1149	EM1237 *hfq*-3xFLAG-kan	This study
KP1150	EM1055 *hfq*-3xFLAG-kan	EM1055 + P1(KP1149)
KP1782	EM1055 *hfq*-3xFLAG-kan Δ*ryhB*::cat	KP1150 + P1(EM1238)
KP1776	EM1055 *hfq*Y25D-3xFLAG-kan	This study
KP1783	EM1055 *hfq*Y25D-3xFLAG-kan ΔryhB::cat	KP1776 + P1(EM1238)
KP1152	EM1055 *hfq*-3xFLAG	KP1150 + pCP20
KP1208	EM1237 *rne*-HA-kan	This study
KP1225	EM1055 *rne*-HA-kan	EM1055 + P1(KP1208)
DL6	EM1055 *hfq*-3xFLAG *rne*-HA-kan	KP1152 + P1(KP1225)
DL8	EM1055 *hfq*-3xFLAG *rne*-HA	DL6 + pCP20
DL14	EM1055 *hfq*-3xFLAG *rne*-HA Δ*araB*::kan	DL8 + P1(GD520)
DL15	EM1055 *hfq*-3xFLAG *rne*-HA Δ*araB*	DL14 + pCP20
DL21	EM1055 *hfq*-3xFLAG *rne*-HA Δ*araB* Δ*rybB*::kan	DL15 + P1(GD549)
DL25	EM1055 *hfq*-3xFLAG *rne*-HA Δ*araB* Δ*rybB*	DL21 + pCP20
DL32	EM1055 *rne*-HA Δ*araB* Δ*rybB* Δ*hfq*-722::kan	DL25 + P1(KP1224)
DL23	EM1055 *hfq*-3xFLAG *rne*-HA Δ*araB* Δ*dsrA*::cat	DL15 + P1(JF138)
DL33	EM1055 *rne*-HA Δ*araB* Δ*dsrA*::cat Δ*hfq*-722::kan	DL23 + P1(KP1224)
DL1662	EM1055 *hfq*-3xFLAG *rne*-HA Δ*araB* Δ*sgrs*::tet	DL15 + P1(DL1028)
DL1686	EM1055 *rne*-HA Δ*araB* Δ*sgrs*::tet Δ*hfq*-722::kan	DL1662 + P1(KP1224)
MPC101	EM1055 *hfq*-3XFLAG *rne*-HA Δ*ara714 leu*[+] Δ*ryhB*::tet	Lalaouna et al. 2015a
DL2	EM1055 *rne*-HA Δ*ara714 leu*[+] Δ*ryhB*::tet Δ*hfq*-722::kan	MPC101 + P1(KP1224)
KP501	EM1055 *sodB*$_{130}$*-lacZ*	EM1055 + λpFRΔ-*sodB*$_{130}$
KP1518	EM1055 *sodB*$_{130}$*-lacZ* Δ*hfq*-722::kan	KP501 + P1(KP1224)
KP662	EM1055 Δ*ara714 leu*[+] Δ*ryhB*::cat *sodB*$_{430}$*-lacZ*	Prevost et al. 2011
KP1039	EM1055 Δ*ara714 leu*[+] Δ*ryhB*::cat *sodB*$_{430}$MH-*lacZ*	EM1455 + λpFRΔ-*sodB*$_{430}$MH
KP1750	EM1055 Δ*ara714 leu*[+] Δ*ryhB*::cat *sodB*$_{430}$*-lacZ* Δ*hfq*-722::kan	KP662 + P1(KP1224)
KP1785	EM1055 *hfq*-3xFLAG-kan *sodB*$_{430}$*-lacZ*	KP1150 + λ(KP662)
KP1845	EM1055 *hfq*-3xFLAG-kan Δ*ryhB*::cat *sodB*$_{430}$*-lacZ*	KP1782 + λ(KP662)
KP1787	EM1055 *hfq*Y25D-3xFLAG-kan *sodB*$_{430}$*-lacZ*	KP1776 + λ(KP662)
KP1788	EM1055 *hfq*Y25D-3xFLAG-kan Δ*ryhB*::cat *sodB*$_{430}$*-lacZ*	KP1783 + λ(KP662)
KP663	EM1055 Δ*ara714 leu*[+] Δ*ryhB*::cat SodB$_{430}$-LacZ	Prevost et al. 2011
KP1040	EM1055 Δ*ara714 leu*[+] Δ*ryhB*::cat SodB$_{430}$MH-LacZ	EM1455 + λpRS1551-SodB$_{430}$MH
DL2067	EM1055 Δ*ara714 leu*[+] Δ*ryhB*::cat SodB$_{430}$-LacZ Δ*hfq*-722::kan	KP663 + P1(KP1224)
KP1489	EM1055 SodB$_{430}$-LacZ	EM1055 + λ(KP663)
KP1490	EM1055 SodB$_{430}$MH-LacZ	EM1055 + λ(KP1040)
KP1493	EM1055 SodB$_{430}$-LacZ Δ*ryhB*::cat	KP1489 + P1(EM1238)
KP1494	EM1055 SodB$_{430}$MH-LacZ Δ*ryhB*::cat	KP1490 + P1(EM1238)
KP1789	EM1055 *hfq*-3xFLAG-kan SodB$_{430}$-LacZ	KP1150 + λ(KP663)
KP1790	EM1055 *hfq*-3xFLAG-kan Δ*ryhB*::cat SodB$_{430}$-LacZ	KP1782 + λ(KP663)
KP1791	EM1055 *hfq*Y25D-3xFLAG-kan SodB$_{430}$-LacZ	KP1776 + λ(KP663)
KP1792	EM1055 *hfq*Y25D-3xFLAG-kan Δ*ryhB*::cat SodB$_{430}$-LacZ	KP1783 + λ(KP663)
GD294	EM1055 Δ*ara714 leu*[+] Δ*ryhB*::cat *sdhC*$_{576}$*-lacZ*	EM1455 + λpFRΔ-*sdhC*$_{576}$
GD707	EM1055 Δ*ara714 leu*[+] Δ*ryhB*::cat *sdhC*$_{576}$MH-*lacZ*	EM1455 + λpFRΔ-*sdhC*$_{576}$MH
KP1751	EM1055 Δ*ara714 leu*[+] Δ*ryhB*::cat *sdhC*$_{576}$*-lacZ* Δ*hfq*-722::kan	GD294 + P1(KP1224)
GD410	EM1055 Δ*ara714 leu*[+] Δ*spf*::cat Δ*ryhB*::tet SdhC$_{258}$-LacZ	Desnoyers and Masse 2012

GD394	EM1055 Δ*ara714 leu*$^+$ Δ*spf*::cat Δ*ryhB*::tet SdhC$_{258}$MH-LacZ	Desnoyers and Masse 2012
DL2066	EM1055 Δ*ara714 leu*$^+$ Δ*spf*::cat Δ*ryhB*::tet SdhC$_{258}$-LacZ Δ*hfq*-722::kan	GD410 + P1(KP1224)
KP1491	EM1055 SdhC$_{258}$-LacZ	EM1055 + λ(GD410)
KP1492	EM1055 SdhC$_{258}$MH-LacZ	EM1055 + λ(GD394)
KP1495	EM1055 SdhC$_{258}$-LacZ Δ*ryhB*::cat	KP1491 + P1(EM1238)
KP1496	EM1055 SdhC$_{258}$MH-LacZ Δ*ryhB*::cat	KP1492 + P1(EM1238)
DL1162	EM1055 Δ*ara714 leu+* Δ*dsrA*::cat	EM1451 + P1(JF138)
KP1923	EM1055 Δ*ara714 leu+* Δ*dsrA*::cat Δ*hfq*-722::kan	DL1162 + P1(KP1224)
KK2562	MG1655 *hfq*Y25D	S. Gottesman Lab (Zhang et al. 2013)

**Plasmids**	**Description**	**Reference**
pNM12	pBAD24 derivative (arabinose inducible promoter; Amp^R)	Majdalani et al. 1998
pBAD-*ryhB*	pNM12 + *ryhB* gene (Amp^R)	Masse et al. 2003
pBAD-MS2-*ryhB*	pBAD-MS2 + *ryhB* gene (Amp^R)	Desnoyers and Masse 2012
pGD3	pBAD33 derivative (arabinose inducible promoter; Cm^R)	Desnoyers and Masse 2012
pGD3-*ryhB*	pGD3 + *ryhB* gene (Cm^R)	This study
pKD4	Template for amplification of kanamycin resistance gene (Amp^R)	Datsenko and Wanner 2000
pKD46	Repts, Ampr, Rec recombinase expression vector (Amp^R)	Datsenko and Wanner 2000
pFRΔ	pRS1553 derivative (for transcriptional fusions) (Amp^R)	Repoila and Gottesman 2001
pRS1551	Plasmid for construction of translational fusions (Amp^R)	Simons et al. 1987
pFRΔ-*sodB*$_{130}$-MS2	pFRΔ + *sodB*$_{130}$ + MS2 aptamer + T7 transcription terminator (Amp^R)	This study
pBAD-*dsrA*	pNM12 + *dsrA* gene (Amp^R)	Majdalani et al. 1998
pBAD-MS2-*dsrA*	pBAD-MS2 + *dsrA* gene (Amp^R)	Lalaouna et al. 2015b
pBAD-*sgrS*	pNM12 + *sgrS* gene (Amp^R)	This study
pBAD-MS2-*sgrS*	pBAD-MS2 + *sgrS* gene (Amp^R)	This study
pBAD-*rybB*	pNM12 + *rybB* gene (Amp^R)	Desnoyers and Masse 2012
pBAD-MS2-*rybB*	pBAD-MS2 + *rybB* gene (Amp^R)	Lalaouna et al. 2015a
pCP20	yeast Flp recombinase gene (Amp^R)	Cherepanov and Wackernagel, 1995

Table S2. List of all oligonucleotides used in this study.

**Primers**	**Sequence 5′-3′**	
EM88	TGTAATACGACTCACTATAGGGCGATCAGGAAGACCCTCGC	Fwd T7 trx RyhB (EMSA)
EM90	TGTAATACGACTCACTATAGGATACGCACAATAAGGCTATTGTAC	Fwd T7 trx *sodB*$_{130}$ (EM1055) *sodB*$_{130}$MH (KP1039)
EM107	CCGATGAATTCTAACTGTCCCGAATGAATTGGTC	Fwd *sdhC*$_{576}$ *Eco*RI
EM168	TCACACTTTGCTATGCCATAGC	Fwd in pBAD24 or pGD3
EM194	GCCATAAACTGCCAGGAATTGG	Fwd in pFRΔ or pRS1551
EM195	CGGGCCTCTTCGCTA	Rv in pFRΔ or pRS1551
EM424	GCTAG**GGATCC**TCGATGGTTTCCGCAGAAATG	Rv trx *sodB*$_{130}$(EM1055) sodB$_{130}$MH(KP1039)
EM455	CAGGCTGAAAATCTTCTCTCATC	Rv in pBAD24 or pGD3
EM1082	GCTAGGGATCCACGACAGTAATAACAAAGG	Fwd *sdhC*$_{576}$ *Bam*HI
EM1293	CCCAGGGAATCCTAAGAACAGC	Fwd *sdhC*$_{576}$MH
EM1294	GCTGTTCTTAGGATTCCCTGGG	Rv *sdhC*$_{576}$MH
EM1370	GGCTATTGTACGTATGCAGGCCGGCAATAAAGGAGAGTAGCAATG	Fwd *sodB*$_{430}$MH
EM1371	CATTGCTACTCTCCTTTATTGCCGGCCTGCATACGTACAATAGCC	Rv *sodB*$_{430}$MH
EM1572	GGGGCTCGAGGCGATCAGGAAGACCCTCGC	Fwd *ryhB* in pGD3 *Xho*I
EM1575	**CGTACCCTGATGGTGTACG**ttaCTCGATGGTTTCCGCAGAAA	Rv *sodB*$_{130}$-**MS2** step 1
EM1576	CCCCAAGGGGTTATGCTAG**CAGACCCTGATGGTGTCTGAAAAACGTACCCTGATGGTGTACG**	Rv *sodB*$_{130}$-**MS2**-T7 terminator step 2
EM1577	GCTA**GGGATCC**CAAAAAACCCCTCAAGACCCGTTTAGAGGCCCCAAGGGGTTATGCTAG	Rv *sodB*$_{130}$-MS2-T7 terminator step 3 **BamHI**
EM1689	**GACTACAAAGACCATGACGGTGATTATAAAGATCATGATATCGACTACAAAGATGACGAC-GATAAATAGTAA**GTGTAGGCTGGAGCTGCT	Fwd *hfq*-3xFLAG step 1 (**3xFLAG** + P1 region (pKD4))
EM1690	GGATCGCTGGCTCCCCGTGTAAAAAAACAGCCCGAAACCTTACATATGAATATCCTCCTTAG	Rv *hfq*-3xFLAG step 1-2 (*hfq* + P2 region (pKD4))
EM1691	GCAGAATACTTCCGCGCAACAGGACAGCGAAGAAACCGAA**GACTACAAAGACCATGACGG**	Fwd *hfq*-3XFLAG step 2 (*hfq* + **3xFLAG**)
EM1978	AAAAAAAAAGCCAGCACCCGGCTGGC	Rv trx RyhB (EMSA)
EM2378	CATAGAATTCGGATAAATTGAGAACGAAAGAT	Rv *ryhB* in pGD3 *Eco*RI
EM2674	GACTGAGAATTCGATGAAGCAAGGGGGTGCCC	Fwd MS2-*sgrS* *Eco*RI
EM2675	CATAGCATGCAAAAAAAACCAGCAGGTATAATCTGCTG	Rv MS2-*sgrS* *Sph*I

EM2676	CCATGATGAAGCAAGGGGGTGCCC	Fwd *sgrS* *Msc*I
**Northern Probes**	**Sequence 5′-3′**	
EM470	TAATACGACTCACTATAGGGAGACGTCACTCCAACGCAGCACC	Rv *lacZ* RNA probe with T7 promoter
EM471	GGTCAATCCGCCGTTTGTTCC	Fwd *lacZ* RNA probe
EM1430	CCAGCATTTCTTCCAGCGTTTCAAGTG	*hns* DNA probe
EM1678	GAGCAATGTCGTGCTTTCAGGTTCTCCGCGAGGGTCTTCCTGA	*ryhB* DNA probe
EM1692	CAGAGCATCTTTAGCATATGGTAGTGCAGGTAATTCGAATG	*sodB* DNA probe
EM1696	GAATAACGCCCACATGCTGTTCTTATTATTCCCTGGGGAC	*sdhC* DNA probe
EM1831	GGTTGATGGGCTCCACAAAATGGGGACATCAAAGAAAAGCAGTGGC	*rybB* DNA probe
EM1884	TGAGGGGGTCGGGATGAAACTTGCTTAAGCAAGAAGCACT	*dsrA* DNA probe
EM1986	CCTGCTACCAGCAGAGCTGGGACCAGGAGGGACAGTACTT	*ompC* DNA probe
EM2267	CTGAAAGTACTTTACAACCCGAAGGCCTTCTTCATACACG	16S DNA probe
EM2268	CACACTACCATCGGCGCTACGGCGTTTCACTTCTGAGTTC	5S DNA probe
EM2693	GGGACGCTTAACCAACGCAACCAGCACAACTTCGCTGTCG	*sgrS* DNA probe
EM4255	GTATTCTGCTGGGCGTCGG	Fwd *ptsG* RNA probe
EM4256	TAATACGACTCACTATAGGGAGACTCCGAGTACGCCAGTATCC	Rv *ptsG* RNA probe with T7 promoter
EM4424	GTTATCGCAGGGAGCCACAC	Fwd *rpoS* RNA probe
EM4425	TGTAATACGACTCACTATAGGCCAGGTTGCGTATGTTGAGAAG	Rv *rpoS* RNA probe with T7 promoter
**FISH Probes**	**Sequence 5′-3′**	
RyhB_1	GCGAGGGTCTTCCTGATCG	RyhB
RyhB_4	CCAGCACCCGGCTGGCTAA	RyhB
lacZ	AATGGGATAGGTCACGTTGG	*lacZ*
	GGCAACATGGAAATCGCTGA	
	GCACGATAGAGATTCGGGAT	
	AGCGGATGGTTCGGATAATG	
	CGCGTACATCGGGCAAATAA	
	CATACAGAACTGGCGATCGT	
	GTAGTTCAGGCAGTTCAATC	
	TTGCACCACAGATGAAACGC	
	CACCCTGCCATAAAGAAACT	
	TAACGCCTCGAATCAGCAAC	
	GATCATCGGTCAGACGATTC	
	TATTCGCAAAGGATCAGCGG	
	AAACTGCTGCTGGTGTTTTG	
	AAAATCCATTTCGCTGGTGG	
ptsG	GCAGGTTAGCAAATGCATTC	*pstG*
	CTGCCATAACATGCGATACA	
	GACACCGATCGCAAAAATCA	
	ATGATGCCATAGGCAACAAC	
	CAGGTGTTTAGAGGCGATTT	
	GGCAGCTTAATACGGTAGAA	
	CAGAAATGATCGGCACAAAG	
	ACTGAGAGAAGGTCTGGATT	
	GGTGTATTCACCAATCTGCA	
	CGGTTTTCTGGTTTAGCAGA	
	GCGGAAGATGGTGTAGTAAA	
	TCGCTTTTGCATCTTCAGTC	
	TACATGCGTCGAGGTTAGTA	
	AGTGGTTACGGATGTACTCA	

1.1. Electrophoretic mobility shift assay (EMSA) of target mRNAs and RyhB with purified Hfq.

5′-end-radiolabeled *sodB₁₃₀*, *sodB₁₃₀*MH or RyhB RNA was used to perform EMSA with Hfq. Hfq was purified as described (Prevost, Salvail et al., 2007). Labeled transcripts (0.2 μM) were heated at 90°C or 2 min then slowly cooled until the temperature reaches 37°C. Yeast tRNA was added (0.1 μg/reaction) and RNAs were incubated in the absence or presence of increasing amounts of purified Hfq during 10 min at 37°C in structure buffer (Ambion). After cooling on ice for 1 min, loading buffer was added and then samples were loaded on 5% native polyacrylamide gel (run in TBE 1X).

1.1.1. In vitro RNA synthesis and radiolabelling.

Oligonucleotides used to generate the DNA template for *in vitro* transcription are indicated in Table S2. T7 RNA polymerase (Roche Diagnostics GmbH, Mannheim, Germany) was used for in vitro transcription. Briefly, transcription was performed in T7 transcription buffer, 5 mM NTP (A, C, G, and U), 40 U of RNaseOut (Invitrogen), 20 U of T7 RNA polymerase, and 0.5 μg of DNA templates. After 4 h of incubation at 37°C, the mixture was treated with 2 U of Turbo DNase (Ambion), extracted once with phenol-chloroform, and purified on denaturing acrylamide gel.

To perform 5′-end labeling, transcripts were dephosphorylated with Alkaline Phosphatase, Calf Intestinal (CIP) (New England Biolabs) and 5′-labeled with [32P]-Ɣ-ATP using T4 polynucleotide kinase (New England Biolabs) according to the manufacturer's protocol.

1.1.2. RNA secondary structure probing.

Secondary structure probing was performed on 5′-end-labeled *sodB*₁₃₀ or *sodB*₁₃₀MH RNA (as above). Hfq was purified as described Prévost et al. (2007). Final concentrations of 0.2 μM *sodB*₁₃₀ or *sodB*₁₃₀MH RNA and increasing amounts of purified Hfq were used. Lead acetate probing, ribonuclease T1 ladder, and alkaline (OH) ladder were performed as described (Desnoyers, Morissette et al., 2009). After reactions, samples were heated for 1 min to 90°C and separated on 8% polyacrylamide/7 M urea sequencing gel.

1.1.3. Hfq^{3xFLAG} and HfqY25D^{3xFLAG} construct

Transfer of the 3xFLAG sequence into chromosomal *hfq* gene was achieved by following the PCR-based method of Datsenko and Wanner (2000). First, Flippase recognition target (FRT)-flanked kanamycin resistance cassette was generated by PCR from pKD4 plasmid with a primer carrying extensions sequences corresponding to the 3xFLAG sequence (forward) and another one that carried extensions homologous to *hfq* gene including the stop codon (oligos EM1689-1690). Another PCR using as template the first PCR was done to add the sequence homologous to the end of *hfq* gene before the stop codon with the same reverse primer as the first PCR (oligos EM1691-1690). For Hfq^{3xFLAG}, the resulting PCR product was transformed into EM1237 after induction of *λred*, according to Yu et al (2000), selecting for kanamycin resistance. To obtain HfqY25D^{3xFLAG} the resulting PCR was transformed to *hfq*Y25D, strains containing the pKD46 plasmid using electroporation Uzzau et al. (2001) and selecting for kanamycin resistance. P1 transduction was used to transfer the linked epitope fusion and the antibiotic resistance gene into a WT background (EM1055). Kanamycin resistance cassette was removed using flippase (Flp) encoding helper plasmid pCP20 as described (Datsenko & Wanner, 2000). All constructs were verified by DNA sequencing and are listed in Table S1. Oligos used for generating the constructs are listed in Table S2.

1.2. Transcriptional and translational lacZ fusions.

To generate *sodB*₄₃₀MH-*lacZ* transcriptional and translational fusions, two independent PCR reactions were performed using the *sodB*₄₃₀ fusion as template with these oligos (EM1370-EM195 and EM194-EM1371). The two PCR products were then mixed to serve as template for a third PCR (EM194–EM195, oligos in the pFRΔ or pRS1551 sequence). The

resulting PCR product was then digested by *Bam*HI and *Eco*RI (NEB) and ligated into *Eco*RI/*Bam*HI-digested pFRΔ (for transcriptional fusions) and *Eco*RI/*Bam*HI-digested pRS1551 (for translational fusions).

For the construction of *sdhC*$_{576}$-*lacZ* transcriptional fusion, a PCR fragment was generated with the following oligos, EM107 and EM1082. To generate *sdhC*$_{576}$MH, two independent PCR reactions were performed using the *sdhC*$_{576}$ fusion as template with these oligos (EM1293-EM195 and EM194-EM1294). The two PCR products were then mixed to serve as template for a third PCR (EM194–EM195). This PCR product was digested by *Eco*RI and *Bam*HI and ligated into *Eco*RI/*Bam*HI-digested pFRΔ.

The transcriptional and the translational fusions were delivered in single copy into the bacterial chromosome of different strains, at the λ *att* site as described previously (Simons, Houman et al., 1987). Stable lysogens were screened for single insertion of recombinant λ by PCR (Powell et al., 1994).

1.3. Plasmids

Plasmid pGD3-*ryhB* was generated by a PCR reaction to produce promoter-less *ryhB* fragment with an *Xho*I site upstream and an *Eco*RI site downstream with the following primers (EM1572-EM2378). The resulting PCR product was then digested with *Xho*I and *Eco*RI and ligated into *Xho*I/*Eco*RI-digested pGD3.

Plasmids pFRΔ-*sodB*$_{130}$-MS2 and pFRΔ-*sodB*$_{130}$MH-MS2 contained the *sodB*$_{130}$ or *sodB*$_{130}$MH RNA with the endogenous promotor followed by the MS2 RNA aptamer and the T7 transcription terminator stemloop. Plasmids were generated by performing a PCR reaction using transcriptional fusions (KP662) as template with oligonucleotides (EM194-EM1575). The PCR product served as template for a second PCR reaction with oligonucleotides (EM194-EM1576). To finish, a third PCR reaction was performed with oligonucleotides (EM194-EM1577). Resulting PCR product was digested with *Eco*RI and *Bam*HI and ligated into *Eco*RI/*Bam*HI-digested pFRΔ.

Plasmid pBAD-MS2-*sgrS* was generated by performing a PCR reaction with oligonucleotides (EM2674-EM2675). The resulting PCR product was then digested with *Eco*RI and *Sph*I and ligated into *Eco*RI/*Sph*I-digested pBAD-MS2. Plasmid pBAD-*sgrS* was generated by performing a PCR reaction with oligonucleotides (EM2675-EM2676). The resulting PCR product was then digested with *Msc*I and *Sph*I and ligated into *Msc*I/*Sph*I-digested pNM12.

References

1. Baba T, Ara T, Hasegawa M, Takai Y, Okumura Y, Baba M, Datsenko KA, Tomita M, Wanner BL, Mori H (2006) Construction of Escherichia coli K-12 in-frame, single-gene knockout mutants: the Keio collection. *Mol Syst Biol* 2: 2006.0008
2. Cherepanov PP, Wackernagel W (1995) Gene disruption in Escherichia coli: TcR and KmR cassettes with the option of Flp-catalyzed excision of the antibiotic-resistance determinant. *Gene* 158: 9-14
3. Datsenko KA, Wanner BL (2000) One-step inactivation of chromosomal genes in Escherichia coli K-12 using PCR products. *Proc Natl Acad Sci U S A* 97: 6640-5
4. Desnoyers G, Morissette A, Prevost K, Masse E (2009) Small RNA-induced differential degradation of the polycistronic mRNA iscRSUA. *EMBO J* 28: 1551-61
5. Majdalani N, Cunning C, Sledjeski D, Elliott T, Gottesman S (1998) DsrA RNA regulates translation of RpoS message by an anti-antisense mechanism, independent of its action as an antisilencer of transcription. *Proc Natl Acad Sci U S A* 95: 12462-7
6. Masse E, Escorcia FE, Gottesman S (2003) Coupled degradation of a small regulatory RNA and its mRNA targets in Escherichia coli. *Genes Dev* 17: 2374-83
7. Powell BS, Rivas MP, Court DL, Nakamura Y, Turnbough CL, Jr. (1994) Rapid confirmation of single copy lambda prophage integration by PCR. *Nucleic Acids Res* 22: 5765-6
8. Prevost K, Salvail H, Desnoyers G, Jacques JF, Phaneuf E, Masse E (2007) The small RNA RyhB activates the translation of shiA mRNA encoding a permease of shikimate, a compound involved in siderophore synthesis. *Mol Microbiol* 64: 1260-73
9. Repoila F, Gottesman S (2001) Signal transduction cascade for regulation of RpoS: temperature regulation of DsrA. *J Bacteriol* 183: 4012-23
10. Salvail H, Caron MP, Belanger J, Masse E (2013) Antagonistic functions between the RNA chaperone Hfq and an sRNA regulate sensitivity to the antibiotic colicin. *Embo j* 32: 2764-78
11. Simons RW, Houman F, Kleckner N (1987) Improved single and multicopy lac-based cloning vectors for protein and operon fusions. *Gene* 53: 85-96

12. Uzzau S, Figueroa-Bossi N, Rubino S, Bossi L (2001) Epitope tagging of chromosomal genes in Salmonella. *Proc Natl Acad Sci U S A* 98: 15264-9
13. Yu D, Ellis HM, Lee EC, Jenkins NA, Copeland NG, Court DL (2000) An efficient recombination system for chromosome engineering in Escherichia coli. *Proc Natl Acad Sci U S A* 97: 5978-83
14. Zhang A, Schu DJ, Tjaden BC, Storz G, Gottesman S (2013) Mutations in interaction surfaces differentially impact E. coli Hfq association with small RNAs and their mRNA targets. *J Mol Biol* 425: 3678-97
